# Education and Midlife Cognitive Functioning: Differing Associations by Childhood Socioeconomic Status?

**DOI:** 10.1093/geroni/igaf122.2291

**Published:** 2025-12-31

**Authors:** Fabio Bolz, John Warren, Eric Grodsky, Chandra Muller

**Affiliations:** University of Minnesota, United States; University of Minnesota, Minneapolis, Minnesota, United States; University of Wisconsin–Madison, Madison, Wisconsin, United States; The University of Texas at Austin, Austin, Texas, United States

## Abstract

Educational attainment has consistently been identified as an important determinant of later-life cognitive functioning. Recent studies, moreover, suggest that additional aspects of the educational process, particularly school quality, may be related to later-life cognition. Another strand of research has examined the association between childhood conditions and later-life cognitive functioning and found strong associations between childhood socioeconomic status and later-life cognition. A substantial part of this association is often found to be mediated by educational attainment. What is not known, however, is whether the associations between the different aspects of the educational process and later-life cognitive functioning vary by childhood socioeconomic status: Is education more beneficial for the cognitive functioning of individuals from lower or higher socioeconomic backgrounds? Using long-spanning nationally representative longitudinal data from the High School & Beyond (HS&B) cohort study we examine whether the associations between school quality, educational attainment, and midlife cognitive functioning vary by childhood socioeconomic status. HS&B includes prospectively collected data on the high schools that the respondents attended and allows us to construct measures of school quality at the school level. The findings will have implications for our understanding of the life course processes underlying disparities in later-life cognitive functioning. Preliminary results suggest that the associations may not vary by childhood socioeconomic status.

